# Accuracy of Ultrasound in Diagnosis of Pneumothorax: A Comparison between Neonates and Adults—A Systematic Review and Meta-Analysis

**DOI:** 10.1155/2019/5271982

**Published:** 2019-12-03

**Authors:** Hamid Dahmarde, Fateme Parooie, Morteza Salarzaei

**Affiliations:** ^1^Department of Radiology, Faculty of Medicine, Zahedan University of Medical Sciences, Zahedan, Iran; ^2^Students Research Committee, Zabol University of Medical Sciences, Zabol, Iran

## Abstract

**Objective:**

The present systematic review and meta-analysis were conducted to investigate the accuracy of ultrasound in the diagnosis of pneumothorax in neonates and adults.

**Method:**

The searches were conducted by two independent researchers (MS and HD) to find the relevant studies published from 01/01/2009 until the end of 01/01/2019. We searched for published literature in the English language in MEDLINE via PubMed, Embase™ via ovid, the Cochrane Library, and Trip database. For literature published in other languages, we searched national databases (Magiran and SID), KoreaMed, and LILACS, and we searched OpenGrey (http://www.opengrey.eu/) and the World Health Organization Clinical Trials Registry (http://who.int/ictrp) for unpublished literature and ongoing studies. The keywords used in the search strategy were pneumothorax or ultrasound or chest ultrasonography or neonate or adult or aerothorax or sensitivity or specificity or diagnostic accuracy. The list of previous study resources and systematic reviews was also searched for identifying the published studies (MS and HD). Analyses were performed using Meta-Disc 1.4.

**Results:**

In total, 1,565 patients (255 neonates, 1212 adults, and 101 pediatrics suspected of pneumothorax) were investigated in 10 studies. The overall specificity of chest ultrasound in the diagnosis of pneumothorax in both populations of adults and neonates was 85.1% at the confidence interval of 95 percent (95% CI 81.1%–88.5%). At the confidence interval of 95 percent, the sensitivity was 98.6% (95% CI 97.7%–99.2%). The diagnostic odds ratio was 387.72 (95% CI 76.204–1972.7). For the diagnosis of pneumothorax in neonates, the ultrasound sensitivity was 96.7% at the confidence interval of 95 percent (95% CI 88.3%–99.6%). At the confidence interval of 95 percent, the specificity was 100% (95% CI 97.7%–100%). For the diagnosis of pneumothorax in adults, the ultrasound sensitivity was 82.9% at the confidence interval of 95 percent (95% CI 78.3–86.9%). At the confidence interval of 95 percent, the specificity was 98.2% (95% CI 97.0%–99.0%). The diagnostic odds ratio was 423.13 (95% CI 45.222–3959.1). Analyzing studies indicated that the sensitivity of “absence lung sliding” sign for the diagnosis of pneumothorax was 87.2% (95% CI 77.7–93.7), and specificity was 99.4% (95% CI 96.5%–100%). DOR was 556.74 (95% CI 100.03–3098.7). The sensitivity of “lung point” sign for the diagnosis of pneumothorax was 82.1% (95% CI 71.7%–89.8%), and the specificity was 100% (at the confidence interval of 95% CI 97.6%–100%). DOR was 298.0 (95% CI 58.893–1507.8).

**Conclusion:**

The diagnosis of pneumothorax using ultrasound is accurate and reliable; additionally, it can result in timely diagnoses specifically in neonatal pneumothorax. Using this method facilitates the therapy process; lack of ionizing radiation and easy operation are benefits of this imaging technique.

## 1. Introduction

Pneumothorax (PTX), which is a common problem in the ICU, is defined as the presence of air in the pleura space; it can be spontaneous, occurring mainly due to trauma or as a result of pathogenic factors such as central venous catheter, mechanical ventilation, thoracocentesis, and pulmonary biopsy [1, 2]. Several studies have investigated the role of chest ultrasound in certain clinical conditions, such as pneumothorax after trauma [3–7], pneumothorax in the intensive care unit [8], and pneumothorax after intervention [9–11]; there have also been several sporadic reports of spontaneous pneumothorax [12]. Chest ultrasound has turned out to be quite effective in the diagnosis of pneumothorax. Despite the increasing use of daily corticosteroids, surfactants, and less-invasive ventilation, pneumothorax (PTX) still continues to be a common cause of respiratory distress in newborns with severe disease and inappropriate ventilation. At the time of respiratory distress, PTX is associated with an increased risk of intraventricular hemorrhage, chronic pulmonary disease, and death. Early diagnosis, accuracy, and rapid detection are the key to successful emergency treatment and saving the life of the newborns [13]. Pneumothorax can be clinically diagnosed by lowered air flow in the auscultation and hypolucent areas in the lung field in chest ultrasound. Unfortunately, the accuracy of the first diagnosis method is uncertain, especially for premature infants [14]. In the past, the diagnosis of pneumothorax was mainly performed through examining the chest X-ray. Wilson-Costello et al. estimated that an average of 31 radiographs is conducted at the hospital when an LBW newborn is admitted. The safety of exposure to this amount of radiation is still under discussion. In addition, interpreting chest ultrasound varies greatly among the specialists [15]. It has also been indicated that diagnosing a small pneumothorax is difficult during the examination of chest X-ray, especially in the birth of preterm infants and low-birth-weight (LBW) infants [16]. Due to the importance of the topic, X-ray examinations are time-consuming and do not help provide timely diagnosis. Chest ultrasound has been recently quite successful in the diagnosis of pneumothorax in clinical emergency care. Due to high sensitivity and specificity, this technology is used as an alternative to chest X-ray examination in detecting pneumothorax [17, 18]. Recent studies also show that chest ultrasound is a promising diagnostic tool in infants with respiratory distress [19–24]. Chest ultrasound is a fascinating alternative because it does not have ionizing radiation, it is quick and easy to repeat, and it can be interpreted by a nonradiologist. Advanced technology has made ultrasound devices smaller and portable; it has also made hospitalized ultrasound and point-of-care testing possible. The present systematic review and meta-analysis were conducted to investigate the accuracy of ultrasound in the diagnosis of pneumothorax in neonates and adults.

## 2. Methods

Presenting a systematic review and meta-analysis based on PRISMA [25] principles.

### 2.1. Search Methods for Eligible Studies

Searching for the eligible studies was conducted in MEDLINE, Embase™, and CINHAL databases from 01/01/2009 to the end of 01/01/2019 by using the following searching strategy.

The searches were conducted by two independent researchers (MS and HD) to find the relevant studies published from 01/01/2009 until the end of 01/01/2019. We searched for published literature in the English language in MEDLINE via PubMed, Embase™ via Ovid, the Cochrane Library, and Trip database. For literature published in other languages, we searched national databases (Magiran and SID), KoreaMed, and LILACS, and we searched OpenGrey (http://www.opengrey.eu/) and the World Health Organization Clinical Trials Registry (http://who.int/ictrp) for unpublished literature and ongoing studies. To ensure the literature saturation, the list of the included research references or the relevant reviews found by searching was studied (FP). The special search strategies were created using the Health Sciences Librarian website with specialization in systematic review searches using the MESH phrases and open phrases in accordance with the PRESS standards [26]. After finalizing the MEDLINE strategy, the results were compared with the search of the other databases (MS and FP). Similarly, PROSPERO was searched to find the recent or ongoing systematic reviews. The keywords used in the search strategy were Pneumothorax [Mesh] OR—Ultrasound [Mesh] OR—chest ultrasonography [Mesh] OR—Neonate [Mesh] OR—Adult [Mesh] OR—aero thorax [Mesh] OR—sensitivity [Mesh] OR—specificity [Mesh] OR—diagnostic accuracy [Mesh]. The list of previous study resources and systematic reviews was also searched for identifying the published studies (MS and HD). In addition, it was attempted to contact the authors of all studies that met the inclusion criteria and request unpublished data and abstracts (FP).

### 2.2. Eligibility Criteria

The inclusion criteria we used to select articles are as follows: (a) original prospective blinded studies investigating the performance of US for pneumothorax diagnosis; (b) avoided studies that included diseased populations (populations with known pneumothorax); (c) described the diagnostic criteria for pneumothorax on US in clear details; and (d) met quality standards, as assessed by the 14-item Quality Assessment of Diagnostic Accuracy Studies (QUADAS-2) tool.

For the meta-analysis, only articles that published a 2 × 2 table or included data that allowed the construction of a 2 × 2 table were included.

### 2.3. Data Extraction and Risk of Bias Evaluation

The data were extracted for evaluating the characteristics of the participants. The index test included characteristics including special equipment and reference standard (executor of the tests and the interval between tests). The information related to diagnosis accuracy is also extracted. The first reader extracted the data (HD). The second reader confirmed the data (MS), and he would have completed them if they were incomplete.

The risk of bias of every article was evaluated by using QUADAS-2 (a revised tool for quality assessment of diagnostic accuracy studies); four possible domains of bias results are evaluated. The first domain is patient selection (selecting the participants based on sequence or random). The participants of the present study are required to have the test conditions. Thus, the risk of bias is high in the studies; only participants suspected of pneumothorax were selected. The second domain is the index test (wrong interpretation of the index test and accurate explanation of detection threshold). The third domain is reference standard or “golden standard” (99% accuracy, the interpretation without considering the results of the index test). The last domain is flow and timing (describing the patients' receiving the index test, the time interval between index tests, and reference standard). Two reviewers evaluated the article independently with QUADAS-2 criteria (MS, FP). After the independent evaluations, the reviewers discussed the article. Each domain was discussed to achieve a single view. The reliability of the reviewers for each domain was measured by using the *κ*-statistic.

### 2.4. Statistical Analysis

On the basis of the results from the 2 × 2 tables, pooled measures for sensitivity, specificity, diagnostic odds ratio (DOR), and area under the curves (AUC) along with their 95% confidence intervals (CIs) were calculated using DerSimonian Lair methodology [27]. Based on the pooled DOR of each index, test summary receiver-operator curves (sROC) were reconstructed using Moses–Shapiro–Littenberg methodology [28]. The DOR reflects the ability of a test to detect, in this case, pneumothorax. A DOR of 1 indicates that the test has no discriminative power. The higher the DOR, the better the diagnostic ability of the imaging modality. To evaluate heterogeneity between studies, the Cochran *Q* statistic and the *I*^2^ index were used. A substantial *I*^2^ index indicates heterogeneity beyond sampling variation. A metaregression analysis was performed to identify predefined sources of heterogeneity. We constructed the forest plots with freeware Meta-Disc, version 1.4 software (http://www.hrc.es/investigacion/metadisc-en.htm; Ramon y Cajal Hospital, Madrid, Spain) [29]. The data related to the diagnostic accuracy of ultrasound were collected for providing a complete analysis. Then, for each of the categories, some studies were meta-analyzed; these studies had high and low risk of bias of participant selection (based on QUADAS-2 criteria).

## 3. Results

### 3.1. Study Selection

**Figure 1:**
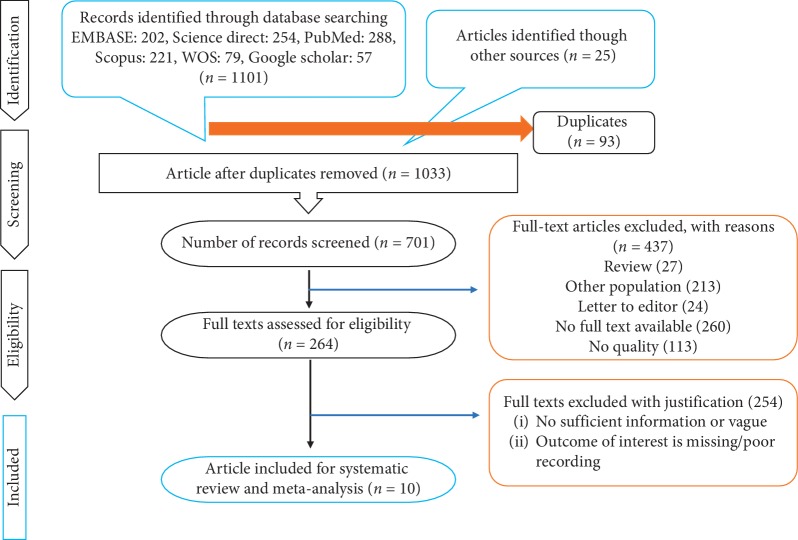
PRISMA flow diagram.

Based on the searching strategy, as many as 1033 studies were selected. After analyzing the correspondence of the studies with the required criteria, 10 studies were selected for the final review (Figure 1).

### 3.2. Characteristics of the Studies

The required characteristics of each selected study have been indicated in Table 1. In total, 1568 patients (255 neonates, 1212 adults, and 101 pediatrics suspected of pneumothorax) were investigated in 10 studies. From these 10 studies, as many as 9 studies (90%) were prospective studies, and 1 study (10%) was a retrospective study. The investigated population was neonates and adults suspected of pneumothorax. Out of 10 studies, 4 were conducted in neonates [13, 18, 30, 31], 2 were conducted in pediatrics [32, 37], and the remained studies were in adult population [33–36]. From 10 studies included, 4 were from Italy [18, 30, 31, 33], 2 from Iran [34, 35], and India [36], Taiwan [37], China [13], and Poland [32], each had 1 included study. (Table 1)

### 3.3. Risk of Bias

**Figure 2:**
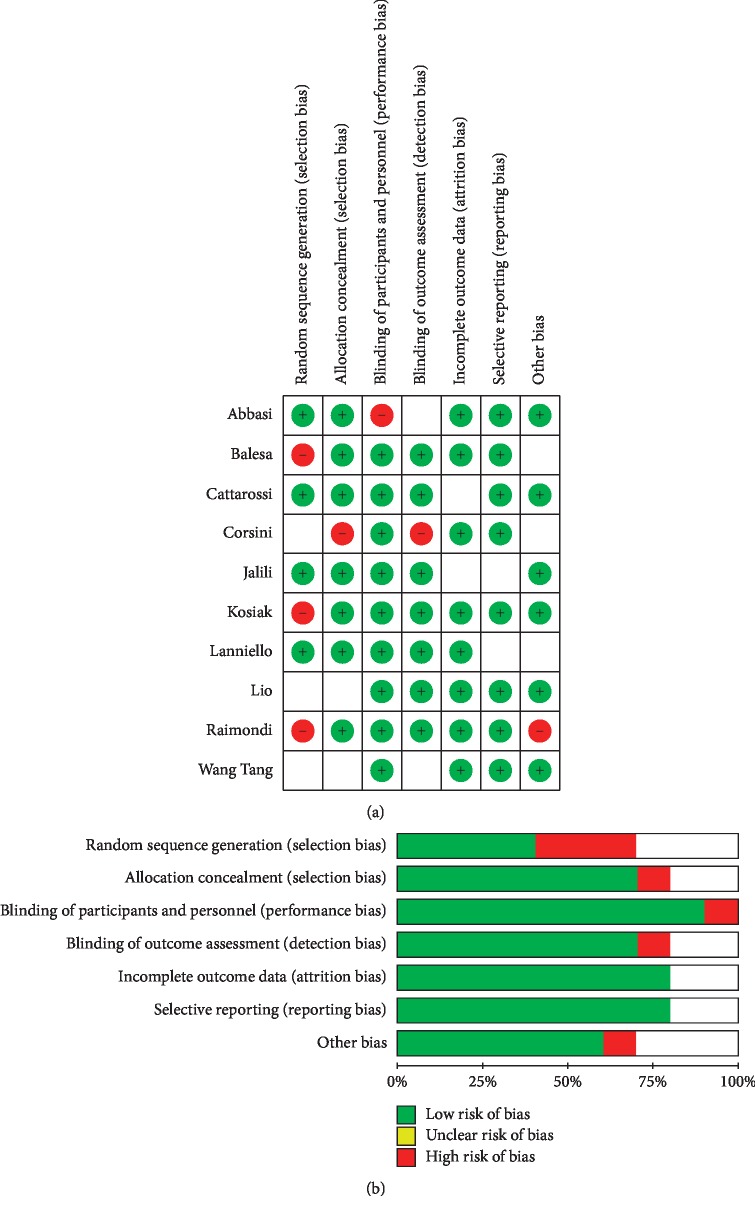
The risk of bias in the studies conducted was measured by using the QUADAS-2 tool. The risk of bias shown in equation b of the above image model (ultrasound) of each diagram indicates the number and percentage of studies with high (red), medium (yellow), and low (green) risk of bias in four groups of the QUADAS-2 tool.

The findings of QUADAS-2 assessment have been indicated in Figure 2.

### 3.4. Synthesis of Results

#### 3.4.1. Overall Meta-Analysis

**Figure 3:**
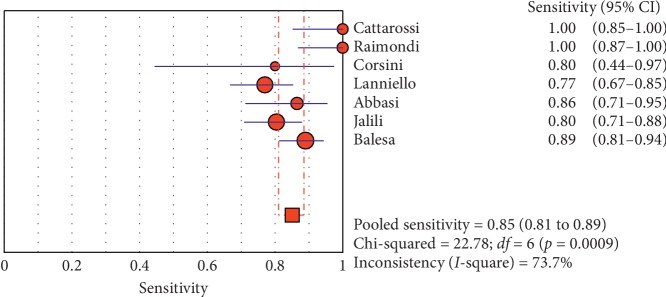
Sensitivity of ultrasound in diagnosis of pneumothorax in studies that included neonates and adults. The Forest plot of sensitivity was reported in each study. Each study is identified by the name of the first author and year of publication, with circles representing individual study point estimates, size of each circle indicating relative contribution to data pooling (inverse variance weighting), horizontal lines indicating 95% CIs, and dashed vertical lines representing 95% CIs for pooled sensitivity and specificity.

**Figure 4:**
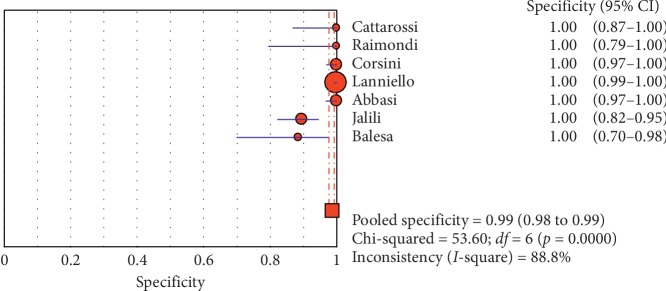
Specificity of ultrasound in diagnosis of pneumothorax in studies that included neonates and adults. Forest plots of specificity reported in each study. Each study is identified by the name of the first author and year of publication, with circles representing individual study point estimates, size of each circle indicating relative contribution to data pooling (inverse variance weighting), horizontal lines indicating 95% CIs, and dashed vertical lines representing 95% CIs for pooled specificity.

**Figure 5:**
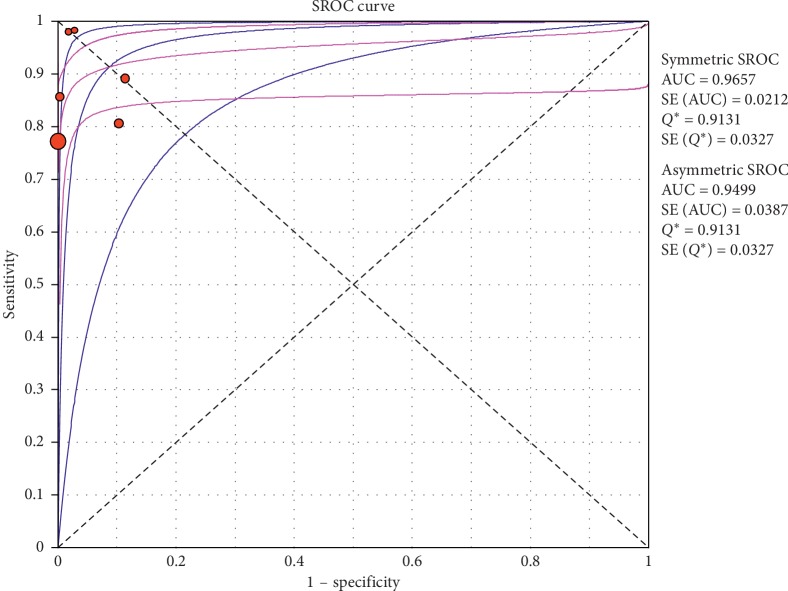
Summary-ROC (SROC) curve for diagnostic accuracy of ultrasound in diagnosis of pneumothorax in adults and neonates. Size of each circle on graph represents the sample size of included study. SE = standard error; *Q*^*∗*^ index = point at which sensitivity and specificity are equal or point closest to the ideal top-left corner of SROC space.

**Figure 6:**
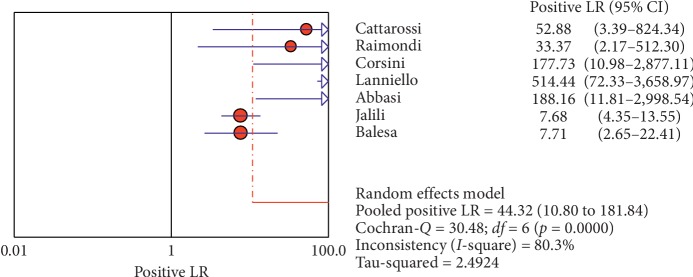
Positive LR of ultrasound in the diagnosis of pneumothorax in studies that included neonates and adults. The Forest plot of positive LR reported in each study. Each study is identified by the name of the first author and year of publication, with circles representing individual study point estimates, size of each circle indicating relative contribution to data pooling (inverse variance weighting), horizontal lines indicating 95% CIs, and dashed vertical lines representing 95% CIs for pooled positive LR.

**Figure 7:**
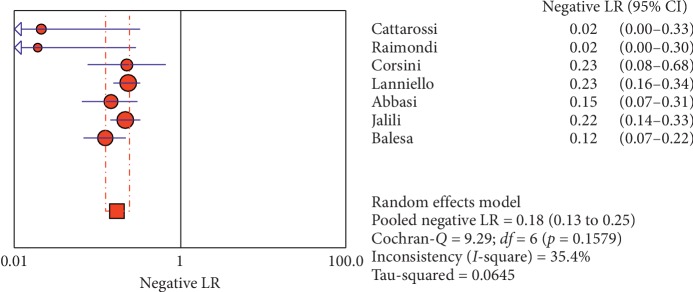
Negative LR of ultrasound in diagnosis of pneumothorax in studies that included neonates and adults. The Forest plot of negative LR reported in each study. Each study is identified by the name of the first author and year of publication, with circles representing individual study point estimates, size of each circle indicating relative contribution to data pooling (inverse variance weighting), horizontal lines indicating 95% CIs, and dashed vertical lines representing 95% CIs for pooled negative LR.

**Figure 8:**
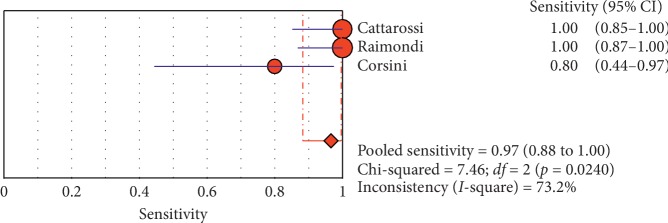
Sensitivity of ultrasound in diagnosis of pneumothorax in studies that included neonates only. The Forest plot of sensitivity reported in each study. Each study is identified by the name of the first author and year of publication, with circles representing individual study point estimates, size of each circle indicating relative contribution to data pooling (inverse variance weighting), horizontal lines indicating 95% CIs, and dashed vertical lines representing 95% CIs for pooled sensitivity and specificity.

**Figure 9:**
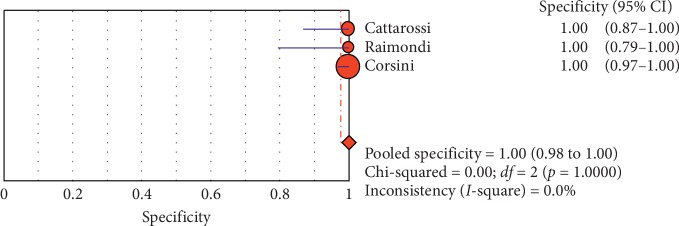
Specificity of ultrasound in diagnosis of pneumothorax in studies that included neonates only. Forest plots of specificity reported in each study. Each study is identified by the name of the first author and year of publication, with circles representing individual study point estimates, size of each circle indicating relative contribution to data pooling (inverse variance weighting), horizontal lines indicating 95% CIs, and dashed vertical lines representing 95% CIs for pooled specificity.

**Figure 10:**
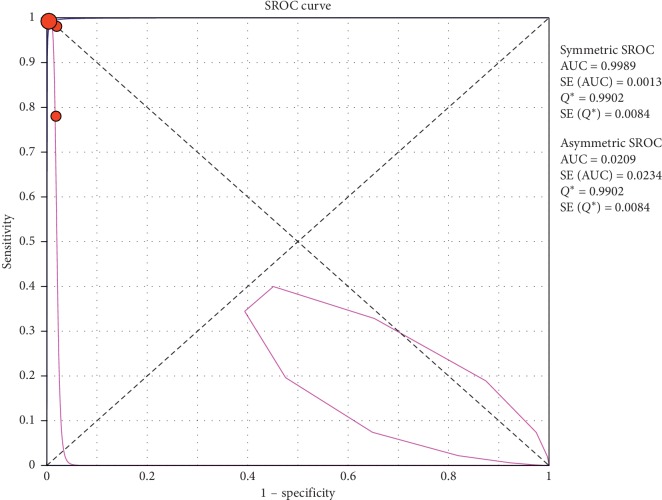
Summary-ROC (SROC) curve for diagnostic accuracy of ultrasound in diagnosis of pneumothorax in neonates only. Size of each circle on graph represents the sample size of included study. SE = standard error; *Q*^*∗*^ index = point at which sensitivity and specificity are equal or point closest to the ideal top-left corner of SROC space.

**Figure 11:**
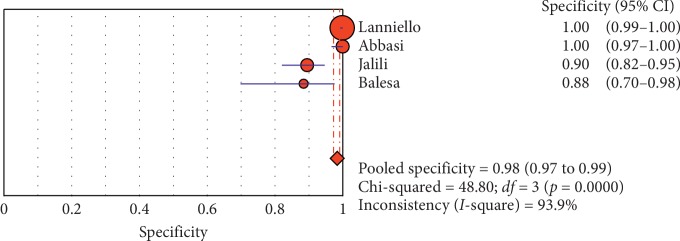
Specificity of ultrasound in diagnosis of pneumothorax in studies that included adults only. Forest plots of specificity reported in each study. Each study is identified by the name of the first author and year of publication, with circles representing individual study point estimates, size of each circle indicating relative contribution to data pooling (inverse variance weighting), horizontal lines indicating 95% CIs, and dashed vertical lines representing 95% CIs for pooled specificity.

**Figure 12:**
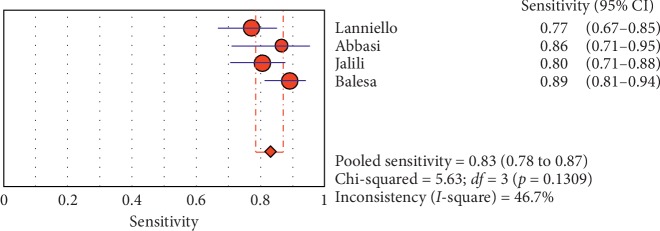
Sensitivity of ultrasound in diagnosis of pneumothorax in studies that included adults only. The Forest plot of sensitivity reported in each study. Each study is identified by the name of the first author and year of publication, with circles representing individual study point estimates, size of each circle indicating relative contribution to data pooling (inverse variance weighting), horizontal lines indicating 95% CIs, and dashed vertical lines representing 95% CIs for pooled sensitivity and specificity.

**Figure 13:**
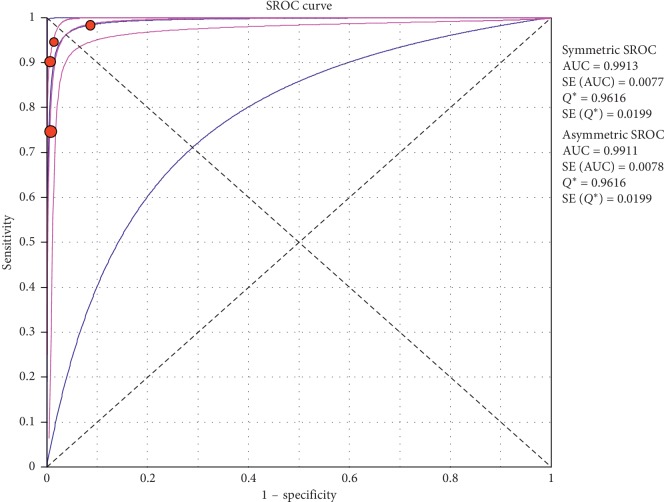
Summary-ROC (SROC) curve for diagnostic accuracy of ultrasound in diagnosis of pneumothorax in adults only. Size of each circle on graph represents the sample size of included study. SE = standard error; *Q*^*∗*^ index = point at which sensitivity and specificity are equal or point closest to ideal top-left corner of SROC space.

The overall specificity of chest ultrasound in diagnosis of pneumothorax in both populations of adults and neonates was 85.1% at the confidence interval of 95 percent (95% CI 81.1%–88.5%). At the confidence interval of 95 percent, the sensitivity was 98.6% (95% CI 97.7%–99.2%). The diagnostic odds ratio was 387.72 (95% CI 76.204–1972.7) showing a relatively high accuracy of chest ultrasound in diagnosing pneumothorax in neonates and adults. The SROC plot showed a summary of estimated sensitivity and specificity and the area under the SROC curve of chest ultrasonography in diagnosing pneumothorax in neonates and adults (Table 2, Figures 3–5). For pneumothorax diagnosis in neonates, the ultrasound sensitivity was 96.7% at the confidence interval of 95 percent (95% CI 88.3%–99.6%). At the confidence interval of 95 percent, the specificity was 100% (95% CI 97.7%–100%). The diagnostic odds ratio was 1343.1 (95% CI 167.20–10788.9) showing a real high accuracy of chest ultrasound in diagnosing pneumothorax in neonates. The SROC plot showed a summary of estimated sensitivity and specificity and the area under the SROC curve of chest ultrasonography in diagnosing pneumothorax in neonates (Table 3, Figures 6–10). For pneumothorax diagnosis in adults, the ultrasound sensitivity was 82.9% at the confidence interval of 95 percent (95% CI 78.3–86.9%). At the confidence interval of 95 percent, the specificity was 98.2% (95% CI 97.0%–99.0%). The diagnostic odds ratio was 423.13 (95% CI 45.222–3959.1), showing lower accuracy of chest ultrasound in diagnosing pneumothorax in adults compared with neonates. The SROC plot showed a summary of estimated sensitivity and specificity and the area under the SROC curve of chest ultrasonography in diagnosing pneumothorax in adults (Table 4) (Figures 11–13).

#### 3.4.2. Subgroup Analysis (Sensitivity and Specificity of Different Ultrasound Manifestations)

**Figure 14:**
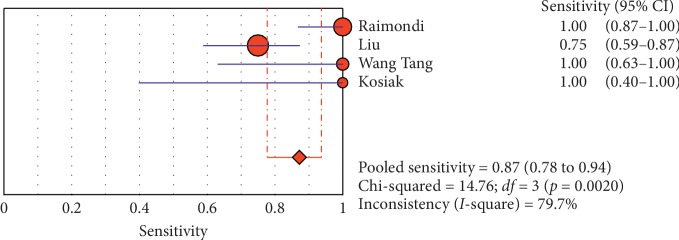
Sensitivity of the “absence of lung sliding” sign in ultrasound in the diagnosis of pneumothorax. The Forest plot of sensitivity reported in each study. Each study is identified by the name of the first author and year of publication, with circles representing individual study point estimates, size of each circle indicating relative contribution to data pooling (inverse variance weighting), horizontal lines indicating 95% CIs, and dashed vertical lines representing 95% CIs for pooled sensitivity and specificity.

**Figure 15:**
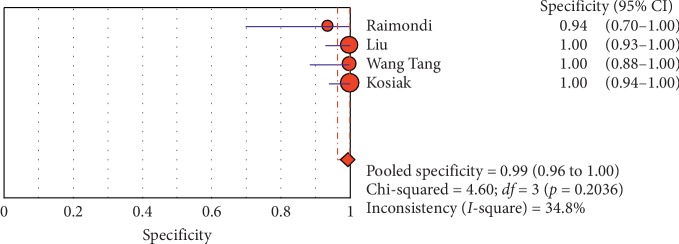
Specificity of the “absence of ling sliding” sign in ultrasound in diagnosis of pneumothorax. Forest plots of specificity reported in each study. Each study is identified by the name of the first author and year of publication, with circles representing individual study point estimates, size of each circle indicating relative contribution to data pooling (inverse variance weighting), horizontal lines indicating 95% CIs, and dashed vertical lines representing 95% CIs for pooled specificity.

**Figure 16:**
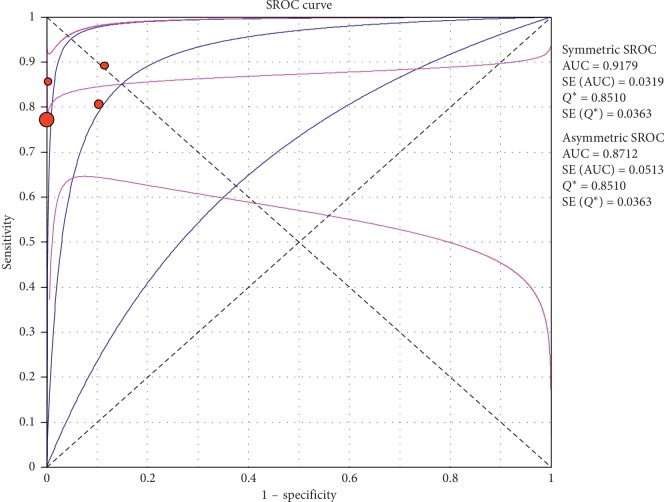
Summary-ROC (SROC) curve for diagnostic accuracy of the absence of lung sliding sign in ultrasound in diagnosis of pneumothorax. Size of each circle on graph represents the sample size of included study. SE = standard error; *Q*^*∗*^ index = point at which sensitivity and specificity are equal or point closest to the ideal top-left corner of SROC space.

**Figure 17:**
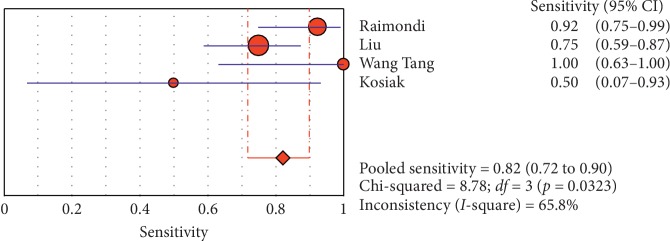
Sensitivity of the “lung point” sign in ultrasound in diagnosis of pneumothorax. The Forest plot of sensitivity reported in each study. Each study is identified by the name of the first author and year of publication, with circles representing individual study point estimates, size of each circle indicating relative contribution to data pooling (inverse variance weighting), horizontal lines indicating 95% CIs, and dashed vertical lines representing 95% CIs for pooled sensitivity and specificity.

**Figure 18:**
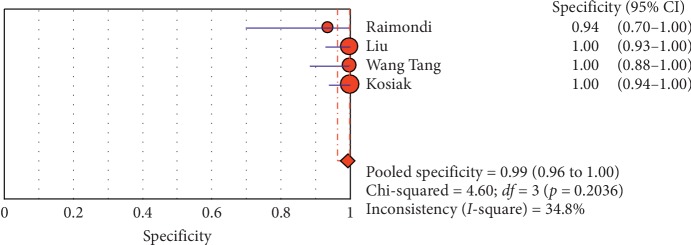
Specificity of the “lung point” sign in ultrasound in diagnosis of pneumothorax. Forest plots of specificity reported in each study. Each study is identified by the name of the first author and year of publication, with circles representing individual study point estimates, size of each circle indicating relative contribution to data pooling (inverse variance weighting), horizontal lines indicating 95% CIs, and dashed vertical lines representing 95% CIs for pooled specificity.

**Figure 19:**
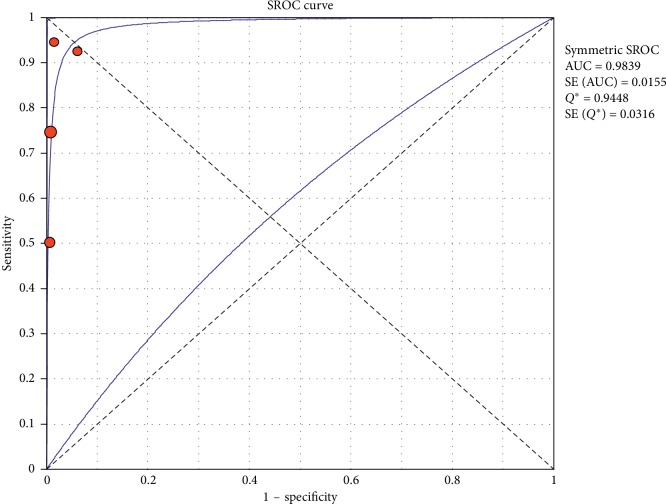
Summary-ROC (SROC) curve for diagnostic accuracy of lung point sign in ultrasound in diagnosis of pneumothorax. Size of each circle on graph represents the sample size of included study. SE = standard error; *Q*^*∗*^ index = point at which sensitivity and specificity are equal or point closest to the ideal top-left corner of SROC space.

Analyzing studies indicated that the sensitivity of the “absence lung sliding” sign for diagnosis of pneumothorax was 87.2 (95% CI 77.7–93.7) and the specificity was 99.4 (at the confidence interval of 95% CI 96.5%–100%). DOR was 556.74 (95% CI 100.03–3098.7) showing a very high accuracy of the “absence lung sliding” sign in diagnosing pneumothorax. The SROC plot showed a summary of estimated sensitivity and specificity and the area under the SROC curve of the “absence lung sliding” sign in diagnosing pneumothorax (Table 5, Figures 14–16). Analyzing studies indicated that the sensitivity of the “lung point” sign for diagnosis of pneumothorax was 82.1% (95% CI 71.7%–89.8%) and the specificity was 100% (at the confidence interval of 95% CI 97.6%–100%). DOR was 298.0 (95% CI 58.893–1507.8) showing a high accuracy of “lung point” sign in diagnosing pneumothorax. The SROC plot showed a summary of estimated sensitivity and specificity and the area under the SROC curve of the “lung point” sign in diagnosing pneumothorax (Table 6, Figures 17–19).

## 4. Discussion

The diagnosis of pneumothorax is generally accompanied by a combination of symptoms and physical examination and is confirmed by chest radiography or CT scan [6]. Late radiography makes it difficult to diagnose pneumothorax due to changes in the patient's condition, distance, or other factors. In addition, chest radiograph is not 100 percent reliable, and misdiagnosis may occur in 30% of all samples of pneumothorax [38]. Earlier studies presented X-rays to be more sensitive to the US, but further research has suggested that the US is more accurate in detecting pneumothorax [39] probably because of newer devices and high-frequency probes. In a survey and meta-analysis in 2014, Ebrahimi et al. revealed the sensitivity and specificity of chest ultrasonography to be 0.87 (95% CI: 0.81–0.92; *I*^2^ = 88.89; *P* < 0.001) and 0.99 (95% CI: 0.98–0.99; *I*^2^ = 86.46, *P* < 0.001), respectively [40]. In the past six years, at least two systematic review and meta-analytic papers and several overview articles have examined the diagnostic accuracy of chest ultrasound in detecting pneumothorax in adults and children [41–44], but none of them have assessed the accuracy of this diagnostic tool in neonates. The present study is the first systematic review and meta-analysis research conducted to evaluate the accuracy of chest ultrasound in the diagnosis of pneumothorax in neonates; it is also the first study to compare the diagnostic accuracy of chest ultrasound in detecting pneumothorax in adults and neonates, as well as the first systematic review in assessing the accuracy, sensitivity, and specificity of different ultrasound manifestations in the diagnosis of pneumothorax. According to the results of the present meta-analysis, the sensitivity, specificity, and odds ratio of the US in the diagnosis of pneumothorax in the general population (adults and neonates) were 98.6% (97.7%–99.2%), 85.1% (88.18%–88.5%), and 387.72% (76.204–1972.7), respectively, while the sensitivity, specificity, and odds ratio of the US in the diagnosis of pneumothorax in neonates were 96.7% (88.3%–99.6%), 100% (97.7%–100%), and 1343.1% (167.20–10788.9), respectively; additionally, the sensitivity, specificity, and odds ratio of the US in the diagnosis of pneumothorax in adults were 82.9% (78.3–86.9%), 98.2% (97.0%–99.0%), and 423.13% (45.222–3959.1), respectively, indicating a higher sensitivity, specificity, and odds ratio of US diagnostic ability for detecting pneumothorax in neonates compared with adults. This issue is of great importance, as neonatal intensive care studies have shown that pneumothorax is hazardous and requires rapid integration of chest tube according to clinical evaluations. The importance of ultrasound presentations is of considerable importance in the present study; “absence of lung sliding” was estimated as the most sensitive ultrasound marker for the diagnosis of pneumothorax with the sensitivity and specificity of 87.2 (77.7–93.7) and 99.4% (96.5%–100%), respectively, and the diagnostic odds ratio of 556.74% (100.03–3098.7), while the sensitivity, specificity, and diagnostic odds ratio of the “lung point” sign was 82.1% (71.7–89.8), 100% (97.6%–100%), and 298 (58.893–1507.8), respectively. Lichtenstein and Mauriat also found that presence of lung sliding or B line can rule out pneumothorax, and presence of lung point represents the diagnosis of pneumothorax [45]. The results of our study confirm the findings of the meta-analysis by Ding et al. The sensitivity and specificity turned out to be 0.88 (0.91–0.85) and 0.99 (0.99–0.98), respectively, for ultrasound in the diagnosis of pneumothorax and 0.52 (0.49–0.55) and 1.00 (1.00–1.00) for CXR [46]; however, the rate of sensitivity found in the present study turned out to be higher than the results of the study of Rajabi et al., which had analyzed 13 studies and showed a sensitivity of 78.6% and a diagnostic accuracy of 98.4% for CUS, while 39.8% and 99.3% were the values obtained for CXR in terms of sensitivity and diagnostic accuracy, respectively [16]. Alrajhi et al. also in their meta-analysis reported a sensitivity of 90.9% and a specificity of 98.2% for the US in detecting pneumothorax [41]. The main limitations of the present study were as follows: it must be kept in mind that the test characteristics are only part of the evaluation of the diagnostic test function, and the extent of each test depends on its effect on the patient. Other important factors such as test-induced damage (in this case, exposure to unnecessary procedures for the treatment of pneumothorax or exposure to ionization radiation), physician's perception and confidence in the test results, as well as the ability to make medical decisions based on the results were not considered in the present research. The nature of the CUS accuracy operator is one of the factors affecting the course and implementation of meta-analysis. The operator training quality is another factor that has not been taken into account in various studies. In addition, the heterogeneity of studies was another issue. Hence, the random effects model was used to provide more accurate results.

## 5. Conclusion

The diagnosis of pneumothorax using ultrasound is accurate and reliable; additionally, it can result in timely diagnoses specifically in neonatal pneumothorax. Using this method facilitates the therapy process; lack of ionizing radiation and easy operation are benefits of this imaging technique.

Table 7 shows the quality of the articles that is calculated using a checklist which includes 5 criteria. Based on these 8 criteria, articles were scored and then classified to three different qualities including good quality (score more than 6), average quality (score 3–6), and weak quality (score below 3). Two studies had good quality. The remained studies were in average quality.

## Figures and Tables

**Table 1:** Summary of included studies.

Author	Year	Country/province	Sampling method	Study design	Study duration	Setting	Mode of data collection	Participants	Age mean ± SD or range	Study population
Cattarossi et al. [30]	2016	Italy	Convenience	Prospective	36 months	Hospital (NICU)	Medical records	49	36 ± 5 weeks	Neonates with respiratory distress
Raimondi et al. [18]	2016	Italy	Convenience	Prospective	12 months	Hospital (NICU)	Medical records	42	31 ± 3.5 weeks	Neonates
Corsini et al. [31]	2018	Italy	Convenience	Prospective	15 months	Hospital (NICU)	Medical records	124	33 ± 5 weeks	Neonates with respiratory distress
Kosiak [32]	2013	Poland	Convenience	Prospective	12 months	Clinic of pediatrics	Medical records	63	1–17 years	Pediatric for whom a central venous catheter was placed in the subclavian vein
Ianniello [33]	2013	Italy	Convenience	Retrospective	24 months	Emergency Department	Medical records	736	16–68	Unstable adult patients
Abbasi et al. [34]	2013	Iran	Convenience	Prospective	1 month	Emergency department	Medical records	153	>16	Adult trauma patients included suspected of having posttraumatic pneumothorax
Jalli et al. [35]	2013	Iran	Convenience	Prospective	26 months	Hospital	Medical records	197	N/A	Patients with pneumothorax
Balesa et al. [36]	2015	India	Convenience	Prospective	29 months	Hospital	Medical records	126	2 months to 88 years	Patients with clinical and/or radiographic suspicion of pneumothorax
Chia-Wang and Kai-Sheng [37]	2014	Taiwan	Convenience	Prospective	24 months	Hospital	Medical records	38	15–18 years	Patients less than 18 years of age admitted with chest pain and/or dyspnea
Liu et al. [13]	2017	China	Convenience	Prospective	13 months	Hospital (NICU)	Medical records	40	N/A	Newborn infants with severe lung disease

**Table 2:** Accuracy of individual studies: chest ultrasound in characterization of pneumothorax.

Study	Year	Participants	TP	FP	FN	TN	Sensitivity (95% CI)			Specificity (95% CI)		
							95%	Low	Up	95%	Low	Up
Cattarossi	2016	49	23	0	0	26	100	0.852	100	100	0.868	100
Raimondi	2016	42	26	0	0	16	100	0.868	100	100	0.794	100
Corsini	2018	124	8	0	2	114	80	0.444	0.975	100	0.968	100
Ianniello	2013	736	67	1	20	667	77	0.668	0.854	0.999	0.992	100
Abbasi	2013	153	32	0	5	109	865	0.712	0.955	100	00.967	100
Jalli	2013	197	74	11	18	94	804	0.709	.880	0.895	0.820	0.947
Balesa	2015	126	89	3	11	23	89	0.812	0.944	0.885	0.698	0.976
Pooled	—	1427	319	15	56	1049	0.986	0.977	0.992	0.851	0.811	0.885

TP, true positive; FP, false positive; FN, false negative; TN, true negative; 95% CI, 95% confidence interval.

**Table 3:** Accuracy of individual studies: chest ultrasound in characterization of pneumothorax in neonates.

Study	Year	Participants	TP	FP	FN	TN	Sensitivity (95% CI)			Specificity (95% CI)		
							95%	Low	Up	95%	Low	Up
Ianniello	2013	736	67	1	20	667	77	0.668	0.854	0.999	0.992	100
Abbasi	2013	153	32	0	5	109	865	0.712	0.955	100	00.967	100
Jalli	2013	197	74	11	18	94	804	0.709	.880	0.895	0.820	0.947
Balesa	2015	126	89	3	11	23	89	0.812	0.944	0.885	0.698	0.976
Pooled		1212	262	15	54	893	0.829	0.783	0.869	0.982	0.970	0.990

TP, true positive; FP, false positive; FN, false negative; TN, true negative; 95% CI, 95% confidence interval.

**Table 4:** Accuracy of individual studies: chest ultrasound in characterization of pneumothorax in adults.

Study	Year	Participants	TP	FP	FN	TN	Sensitivity (95% CI)			Specificity (95% CI)		
							95%	Low	Up	95%	Low	Up
Cattarossi	2016	49	23	0	0	26	100	0.852	100	100	0.868	100
Raimondi	2016	42	26	0	0	16	100	0.868	100	100	0.794	100
Corsini	2018	124	8	0	2	114	80	0.444	0.975	100	0.968	100
Pooled	—	215	57	0	2	156	0.966	0.883	0.996	100	0.977	100

TP, true positive; FP, false positive; FN, false negative; TN, true negative; 95% CI, 95% confidence interval.

**Table 5:** Accuracy of individual studies: absence of lung sliding in characterization of pneumothorax.

Study	Year	Participants	TP	FP	FN	TN	Sensitivity (95% CI)			Specificity (95% CI)		
							95%	Low	Up	95%	Low	Up
Raimondi	2016	42	24	0	2	16	0.923	0.749	0.991	100	0.794	100
Liu	2017	90	30	0	10	50	0.750	0.588	0.873	100	0.929	100
Wang Tang	2014	38	8	0	0	30	100	0.631	100	100	0.884	100
Kosiak	2013	63	2	0	2	59	0.50	0.068	0.932	100	0.939	100
Pooled	—	233	64	—	14	155	0.821	0.717	0.898	100	0.976	100

TP, true positive; FP, false positive; FN, false negative; TN, true negative; 95% CI, 95% confidence interval.

**Table 6:** Accuracy of individual studies: lung point in characterization of pneumothorax.

Study	Year	Participants	TP	FP	FN	TN	Sensitivity (95% CI)			Specificity (95% CI)		
							95%	Low	Up	95%	Low	Up
Raimondi	2016	42	26	1	0	15	100	0.868	100	0.938	0.698	0.998
Liu	2017	90	30	0	10	50	0.750	0.588	0.873	100	0.929	100
Wang Tang	2014	38	8	0	0	30	100	0.631	100	100	0.884	100
Kosiak	2013	63	4	0	0	59	100	0.398	100	100	0.939	100
Pooled	—	228	68	1	10	154	0.872	0.777	0.937	0.994	0.965	100

TP, true positive; FP, false positive; FN, false negative; TN, true negative; 95% CI, 95% confidence interval.

**Table 7:** Quality of included articles.

First author	Country	Year	Sample size	US TP, FP	US TN, FN	CXR sensitivity	CXR specificity	Lung point sen and spe	Lung-sliding absence sen and spe	B-line absence sen and spe
Cattarossi	Italy	2016	^*∗*^	^*∗*^	^*∗*^	^*∗*^	^*∗*^			
Raimondi	Italy	2016	^*∗*^	^*∗*^	^*∗*^			^*∗*^	^*∗*^	^*∗*^
Corsini	Italy	2018	^*∗*^	^*∗*^	^*∗*^					
Kosiak	Poland	2013	^*∗*^			^*∗*^	^*∗*^	^*∗*^	^*∗*^	^*∗*^
Ianniello	Italy	2013	^*∗*^	^*∗*^	^*∗*^					
Abbasi	Iran	2013	^*∗*^	^*∗*^	^*∗*^	^*∗*^	^*∗*^			
Jalli	Iran	2013	^*∗*^	^*∗*^	^*∗*^	^*∗*^	^*∗*^			
Balesa	India	2015	^*∗*^	^*∗*^	^*∗*^			^*∗*^	^*∗*^	
Wang Tang	Taiwan		^*∗*^					^*∗*^	^*∗*^	
Liu	China		^*∗*^					^*∗*^	^*∗*^	

sen: sensitivity; spe: specificity.

## Abbreviations

US: Ultrasound
CUS: Chest ultrasound
PTX: Pneumothorax
CXR: Chest X-ray
LBW: Low-birth-weight
ICU: Intensive care unit

## References

[1] Strange C. (1999). Pleural complications in the intensive care unit. *Clinics in Chest Medicine*.

[2] de Latorre F. J., Klamburg J., Leon C., Soler M., Rius J., Rius J. (1977). Incidence of pneumothorax and pneumomediastinum in patients with aspiration pneumonia requiring ventilatory support. *Chest*.

[3] Nandipati K. C., Allamaneni S., Kakarla R. (2011). Extended focused assessment with sonography for trauma (EFAST) in the diagnosis of pneumothorax: experience at a community based level I trauma center. *Injury*.

[4] Gillman L. M., Ball C. G., Panebianco N., Al-Kadi A., Kirkpatrick A. W. (2009). Clinician performed resuscitative ultrasonography for the initial evaluation and resuscitation of trauma. *Scandinavian Journal of Trauma, Resuscitation and Emergency Medicine*.

[5] Stone M. (2008). Ultrasound diagnosis of traumatic pneumothorax. *Journal of Emergencies, Trauma and Shock*.

[6] Soldati G., Testa A., Sher S., Pignataro G., La Sala M., Silveri N. G. (2008). Occult traumatic pneumothorax: diagnostic accuracy of lung ultrasonography in the emergency department. *Chest*.

[7] Donmez H., Tokmak T. T., Yildirim A. (2012). Should bedside sonography be used first to diagnose pneumothorax secondary to blunt trauma?. *Journal of Clinical Ultrasound*.

[8] Lichtenstein D. A., Menu Y. (1995). A bedside ultrasound sign ruling out pneumothorax in the critically ill: lung sliding. *Chest*.

[9] Reissig A., Kroegel C. (2005). Accuracy of transthoracic sonography in excluding post-interventional pneumothorax and hydropneumothorax. Comparison to chest radiography. *European Journal of Radiology*.

[10] Targhetta R., Bourgeois J.-M., Chavagneux R., Balmes P. (1992). Diagnosis of pneumothorax by ultrasound immediately after ultrasonically guided aspiration biopsy. *Chest*.

[11] Sartori S., Tombesi P., Trevisani L., Nielsen I., Tassinari D., Abbasciano V. (2007). Accuracy of transthoracic sonography in detection of pneumothorax after sonographically guided lung biopsy: prospective comparison with chest radiography. *American Journal of Roentgenology*.

[12] Volpicelli G., Audino B. (2011). The double lung point: an unusual sonographic sign of juvenile spontaneous pneumothorax. *The American Journal of Emergency Medicine*.

[13] Liu J., Chi J.-H., Ren X.-L. (2017). Lung ultrasonography to diagnose pneumothorax of the newborn. *The American Journal of Emergency Medicine*.

[14] Volpicelli G. (2011). Sonographic diagnosis of pneumothorax. *Intensive Care Medicine*.

[15] Wilson-Costello D., Rao P. S., Morrison S., Hack M. (1996). Radiation exposure from diagnostic radiographs in extremely low birth weight infants. *Pediatrics*.

[16] Alrajab S., Youssef A. M., Akkus N. I., Caldito G. (2013). Pleural ultrasonography versus chest radiography for the diagnosis of pneumothorax: review of the literature and meta-analysis. *Critical Care*.

[17] Volpicelli G., Elbarbary M., Blaivas M. (2012). International evidence-based recommendations for point-of-care lung ultrasound. *Intensive Care Medicine*.

[18] Raimondi F., Cattarossi L., Copetti R. (2014). International perspectives: point-of-care chest ultrasound in the neonatal intensive care unit: an Italian perspective. *NeoReviews*.

[19] Rachuri H., Oleti T. P., Murki S., Subramanian S., Nethagani J. (2017). Diagnostic performance of point of care ultrasonography in identifying the etiology of respiratory distress in neonates. *The Indian Journal of Pediatrics*.

[20] Chen S. W., Fu W., Liu J., Wang Y. (2017). Routine application of lung ultrasonography in the neonatal intensive care unit. *Medicine (Baltimore)*.

[21] Copetti R., Cattarossi L. (2007). The “double lung point”: an ultrasound sign diagnostic of transient tachypnea of the newborn. *Neonatology*.

[22] Vergine M., Copetti R., Brusa G., Cattarossi L. (2014). Lung ultrasound accuracy in respiratory distress syndrome and transient tachypnea of the newborn. *Neonatology*.

[23] Bober K., Swietliński J. (2006). Diagnostic utility of ultrasonography for respiratory distress syndrome in neonates. *Medical Science Monitor*.

[24] Copetti R., Cattarossi L., Macagno F., Violino M., Furlan R. (2008). Lung ultrasound in respiratory distress syndrome: a useful tool for early diagnosis. *Neonatology*.

[25] Moher D., Liberati A., Tetzlaff J., Altman D. G., The PRISMA Group (2009). Preferred reporting items for systematic reviews and meta-analyses:the PRISMA statement. *PLoS Medicine*.

[26] Watkinson M., Tiron I. (2001). Events before the diagnosis of a pneumothorax in ventilated neonates. *Archives of Disease in Childhood: Fetal and Neonatal Edition*.

[27] DerSimonian R., Laird N. (1986). Meta-analysis in clinical trials. *Controlled Clinical Trials*.

[28] Moses L. E., Shapiro D., Littenberg B. (1993). Combining independent studies of a diagnostic test into a summary ROC curve: data-analytic approaches and some additional considerations. *Statistics in Medicine*.

[29] Zamora J., Abraira V., Muriel A., Khan K., Coomarasamy A. (2006). Meta-DiSc: a software for meta-analysis of test accuracy data. *BMC Medical Research Methodology*.

[30] Cattarossi L., Copetti R., Brusa G., Pintaldi S. (2016). Lung ultrasound diagnostic accuracy in neonatal pneumothorax. *Canadian Respiratory Journal*.

[31] Corsini I., Parri N., Gozzini E. (2019). Lung ultrasound for the differential diagnosis of respiratory distress in neonates. *Neonatology*.

[32] Kosiak W. (2013). Sonography of iatrogenic pneumothorax in pediatric patients. *Journal of Ultrasonography*.

[33] Ianniello S., Di Giacomo V., Sessa B., Miele V. (2014). First-line sonographic diagnosis of pneumothorax in major trauma: accuracy of e-FAST and comparison with multidetector computed tomography. *La Radiologia Medica*.

[34] Abbasi S., Farsi D., Hafezimoghadam P., Fathi M., Zare M. A. (2013). Accuracy of emergency physician-performed ultrasound in detecting traumatic pneumothorax after a 2-h training course. *European Journal of Emergency Medicine*.

[35] Jalli R., Sefidbakht S., Jafari S. H. (2013). Value of ultrasound in diagnosis of pneumothorax: a prospective study. *Emergency Radiology*.

[36] Balesa J., Rathi V., Kumar S., Tandon A. (2015). Chest sonography in the diagnosis of pneumothorax. *The Indian Journal of Chest Diseases & Allied Sciences*.

[37] Chia-Wang T., Kai-Sheng H. (2013). Bedside sonographic diagnosis of pneumothorax in pediatric patients: a preliminary report. *J Pediatr Resp Dis*.

[38] Rowan K. R., Kirkpatrick A. W., Liu D., Forkheim K. E., Mayo J. R., Nicolaou S. (2002). Traumatic pneumothorax detection with thoracic US: correlation with chest radiography and CT-initial experience. *Radiology*.

[39] Turk F., Kurt A. B., Saglam S. (2010). Evaluation by ultrasound of traumatic rib fractures missed by radiography. *Emergency Radiology*.

[40] Ebrahimi A., Yousefifard M., Kazemi H. M (2014). Diagnostic accuracy of chest ultrasonography versus chest radiography for identification of pneumothorax: a systematic review and meta-analysis. *Tanaffos*.

[41] Alrajhi K., Woo M. Y., Vaillancourt C. (2012). Test characteristics of ultrasonography for the detection of pneumothorax: a systematic review and meta-analysis. *Chest*.

[42] Heuvelings C. C., Bélard S., Familusi M. A., Spijker R., Grobusch M. P., Zar H. J. (2018). Chest ultrasound for the diagnosis of paediatric pulmonary diseases: a systematic review and meta-analysis of diagnostic test accuracy. *British Medical Bulletin*.

[43] Lichtenstein D. A. (2014). Lung ultrasound in the critically ill. *Annals of Intensive Care*.

[44] Shostak E., Brylka D., Krepp J., Pua B., Sanders A. (2013). Bedside sonography for detection of postprocedure pneumothorax. *Journal of Ultrasound in Medicine*.

[45] Lichtenstein D. A., Mauriat P. (2012). Lung ultrasound in the critically ill neonate. *Current Pediatric Reviews*.

[46] Ding W., Shen Y., Yang J., He X., Zhang M. (2011). Diagnosis of pneumothorax by radiography and ultrasonography: a meta-analysis. *Chest*.

